# 2022 revised European recommendations for the coding of the basis of diagnosis of cancer cases in population-based cancer registries

**DOI:** 10.3389/fonc.2023.1250549

**Published:** 2023-12-12

**Authors:** Otto Visser, Beata Kościańska, Florentino Luciano Caetano dos Santos, Francesco Cuccaro, Gonçalo Forjaz, Irmina Maria Michalek, Mohsen Mousavi, Urszula Sulkowska, Carmen Martos, Francesco Giusti

**Affiliations:** ^1^Department of Registration, Netherlands Comprehensive Cancer Organization (IKNL), Utrecht, Netherlands; ^2^Lublin Cancer Registry, Lublin, Poland; ^3^Polish National Cancer Registry, Maria Sklodowska-Curie National Research Institute of Oncology, Warsaw, Poland; ^4^Cancer Registry of Puglia, Section of Health Local Unit Barletta–Andria–Trani, Barletta, Italy; ^5^Westat, Rockville, MD, United States; ^6^Graubünden and Glarus Cancer Registry, Chur, Switzerland; ^7^Mazowiecki Cancer Registry, Maria Sklodowska-Curie National Research Institute of Oncology, Warsaw, Poland; ^8^European Commission, Joint Research Centre, Ispra, Italy

**Keywords:** basis of diagnosis, recommendations, cancer, cancer registration, Europe

## Abstract

The basis of diagnosis recommendations for population-based cancer registries aim to provide a standardized coding tool that reflects the certainty of cancer diagnosis, especially when pathological confirmation is lacking. The proportion of clinical diagnoses serves as an indicator of data quality. Given the evolving nature of diagnostic techniques, regular revision of the basis of diagnosis rules is crucial. To address this, a working group comprising representatives from the steering committee and member registries of the European Network of Cancer Registries was established. The original 1999 recommendations were comprehensively reviewed, resulting in the publication of an updated version. These new recommendations came into effect for incident cancer cases starting from January 1, 2023. The updated recommendations comprise an adapted code list for the basis of diagnosis, optional codes for histology cases, revisions related to flow cytometry, liquid biopsy, and cytogenetic/molecular testing, consolidation of histology codes 6 and 7, introduction of a new code 8 for cytogenetic/molecular confirmation, and establishment of new criteria for registering specific morphology codes in cancers lacking pathological confirmation.

## Introduction

The methods used to diagnose cancer have greatly improved over time. While pathological diagnosis is still the gold standard, an increasing number of cancers, such as hepatocellular carcinoma, can be diagnosed using modern imaging techniques with acceptable certainty without pathological confirmation (1), Imaging techniques are especially relevant for cancer cases which require invasive (and potentially harmful) techniques to obtain a sample for pathological examination, such as tumors of the pancreas, liver, and central nervous system.

The most valid basis of diagnosis is one of the key variables in population-based cancer registries (2). International studies show that there is a large variation in the distribution of the basis of diagnosis of registered cancer cases. For example, in a study of Berrino et al. the proportion of microscopically verified cases ranged from 79% in Poland to 98% in Sweden (3). This may be due to real variation but may also be caused by differences in registration practices or in interpretation of the coding. Clear guidelines for the coding of the basis of diagnosis should reduce these differences in interpretation and contribute to the comparability of the data.

The aim of the Basis of Diagnosis Recommendations by the European Network of Cancer Registries (ENCR) is to provide guidelines to European cancer registries for defining the level of certainty of the diagnosis of cancer (4). This is particularly relevant in the absence of a pathological confirmation of cancer. The proportion of clinical diagnoses (basis of diagnosis codes 1, 2, or 4) is an indicator of the quality of the data of a cancer registry. While a high proportion of clinical diagnoses in a cancer registry may well reflect the extent of the clinical and pathological investigations in the registry area, especially in developing countries, it may also indicate an overestimation of the cancer incidence. For example if non-malignant lesions without pathology are erroneously included in a cancer registry, cancer incidence will be inflated. Besides, cancer survival will be overestimated, as the risk of dying from a non-malignant disease will generally be much lower than from a malignancy.

In registries with a (very) low proportion of clinical diagnoses, there may be an underestimation of cancer incidence due to incomplete notification of clinically and/or radiologically confirmed cancer cases. In many cancer registries, notification of pathologically confirmed cases is better organized than notification of cancer cases with a clinical diagnosis only. Consequently, these cancer registries run the risk of incompleteness of cancers, such as lung cancer, pancreatic cancer, and several hematological malignancies which are not confirmed pathologically in a considerable proportion of cases.

Traditionally, cancer cases without pathological confirmation were coded by cancer registries with an unspecified morphology code according to ICD-O, i.e. 9990/3 in the first edition (5) and 8000/3 as of the second edition (6). For several cancer entities, exceptions were made to this rule, as indicated by the 1999 ENCR recommendations (7). Since then, imaging techniques have improved, and additional techniques have become available, such as molecular diagnostics, which has increased the number of cancer entities which may be diagnosed with reasonable certainty in the absence of pathology. Therefore, these recommendations required further revision.

## Methods

During the summer of 2021, the ENCR initiated a call for expressions of interest from member registries to form a working group (WG) with the purpose of updating the ENCR recommendations on the basis of diagnosis, originally published in 1999. The primary objective of this WG with expertise in cancer registration, epidemiology, pathology and radiotherapy, was to enhance the comparability of incidence and survival data between different European registries and countries. Following the establishment of the WG, a proposal was formulated by one of its members (OV). Subsequently, an online meeting took place on October 27, 2021, during which the proposal was deliberated upon. An amended proposal, agreed upon by all members, was circulated. The draft recommendations were then scrutinized and endorsed by the ENCR Steering Committee (SC) on November 9, 2021. Following the SC’s approval, the recommendations were disseminated to all ENCR members for consultation. Fourteen cancer registries provided feedback, which was subsequently discussed among the WG members. Based on this discussion several modifications were incorporated. On June 8, 2022, the SC granted final approval to the revised recommendations, which were subsequently published on the ENCR website on October 20, 2022. Lastly, on November 30, 2022, a webinar was organized, specifically for registry staff from ENCR institutions, to provide a detailed explanation of the new recommendations.

## Results and discussion

The recommendations (8) include an adapted code list for the basis of diagnosis, as presented in **Table 1**. Additionally, **Table 2** provides optional codes for cases with histology as the basis of diagnosis. The revisions made to the previous version of the recommendations pertain primarily to flow cytometry, liquid biopsy, and cytogenetic and/or molecular testing. Furthermore, the original code 6 (histology of metastasis) has been merged with code 7 (histology of primary tumor) into consolidated code 7, which now includes histology of primary tumor, histology of metastasis, and histology at autopsy. As a result, code 6 is no longer used in the updated recommendations. Additionally, a new code 8 has been introduced for cancer cases with cytogenetic or molecular confirmation of the diagnosis, which was not present in the original recommendations.

**Table 1:** Basis of diagnosis codes.

Code	Description	Criteria
0	Death certificate only (DCO)	Information provided is from a death certificate.
1	Clinical	Diagnosis made before death, but without any of the following (codes 2-8).
2	Clinical investigation	All diagnostic techniques, including X-ray, endoscopy, imaging, ultrasound, exploratory surgery (such as laparotomy), and autopsy, without a tissue diagnosis.
4	Specific tumor markers	Including biochemical and/or immunologic markers that are specific for a tumor site.
5	Cytology	Examination of cells from a primary or secondary site, including fluids aspirated by endoscopy or needle; also includes the microscopic examination of peripheral blood and bone marrow aspirates, immunophenotyping by flow cytometry and a liquid biopsy^#^ in the absence of pathology.
7	Histology	Histologic examination of tissue from the tumor (primary or metastatic), however obtained, including all cutting techniques and bone marrow biopsies; also includes autopsy specimens of the tumor.
8	Cytogenetic and/or molecular testing	Detection of tumor-specific genetic abnormalities or genetic changes in the tumor, including techniques such as karyotyping, FISH (fluorescent *in situ* hybridization), PCR (polymerase chain reaction), DNA sequencing
9	Unknown	

^#^ a liquid biopsy is a sample of blood or another body fluid (liquor, etc.) for the detection of cancer cells or DNA-fragments of these tumor cells.

**Table 2:** Optional codes for cases with histology basis of diagnosis.

Code	Description	Criteria
7.1	Histology of the primary tumor	Histologic examination of tissue from the primary tumor, however obtained, including all cutting techniques and bone marrow biopsies.
7.2	Histology of a metastasis	No histology of the primary tumor
7.3	Histology at autopsy	No histology before autopsy

Furthermore, a compilation of cancers has been created, which may be registered with a specific morphology based on clinical information or clinical investigations when pathology results are unavailable. The list is presented in **Table 3**. In exceptional cases, other specific cancers may be diagnosed through clinical investigations; however, assignment of a specific morphology code should only be performed after careful evaluation by a coding expert from the cancer registry.

**Table 3:** Cancers that may be registered with a specific morphology based on clinical information (basis of diagnosis code 1) or clinical investigations (basis of diagnosis code 2).

Cancer type	Basis of diagnosis code	ICD-O topography code	ICD-O morphology code
Melanoma			
- Melanoma of the skin	1	C44	8720/3
- Melanoma of the eye	1 or 2	C69.0, C69.3, C69.4	8720/3
Solid childhood cancers (age <15 years)			
- Nephroblastoma	2	C64	8960/3
- Hepatoblastoma	2	C22	8970/3
- Retinoblastoma	1 or 2	C69.2	9510/3
Hepatocellular carcinoma	2	C22.0	8170/3
Cholangiocarcinoma	2	C22.1, C24.0, C24.9	8160/3
Non-functioning neuroendocrine tumors (NETs)			
- Non-functioning NET of the pancreas	2	C25.4	8150/3
- Non-functioning NET of the small intestine	2	C17	8240/3
Intraductal papillary mucinous neoplasm (IPMN)	2	C25	8453/2, 8453/3
Sarcoma			
- Sarcoma, NOS	2	^*^	8800/3
- Liposarcoma	2	^*^	8850/3
- Leiomyosarcoma	2	^*^	8890/3
- Angiosarcoma	1^**^ or 2	^*^	9120/3
- Kaposi sarcoma of the skin	1	C44	9140/3
- Osteosarcoma - Chondrosarcoma	2 2	C40, C41 C40, C41	9180/3 9220/3
- Chordoma	2	C41.0	9370/3
Central nervous system (CNS) tumors			
- Mature teratoma, cystic teratoma	2	C71, C75.1, C75.3	9080/0
- Teratoma, NOS	2	C71, C75.1, C75.3	9080/1
- Immature teratoma, malignant teratoma	2	C71, C75.1, C75.3	9080/3
- Hemangioblastoma	2	C71, C72.0	9161/1
- Craniopharyngioma	2	C75.2	9350/1
- Pinealoma	2	C75.3	9360/1
- Pineocytoma	2	C75.3	9361/1
- Pineoblastoma	2	C75.3	9362/3
- Glioma, NOS	2	C71, C72.0	9380/39
- Low grade glioma	2	C71, C72.0	9380/32
- High grade glioma	2	C71, C72.0	9380/33
- Subependymoma	2	C71.5, C71.7	9383/1
- Subependymal giant cell astrocytoma	2	C71.5, C71.7	9384/1
- Choroid plexus papilloma	2	C71.5, C71.7	9390/0
- Atypical choroid plexus papilloma	2	C71.5, C71.7	9390/1
- Choroid plexus carcinoma	2	C71.5, C71.7	9390/3
- Ependymoma	2	C71.5, C71.7, C72.0	9391/3
- Anaplastic ependymoma	2	C71.5, C71.7, C72.0	9392/3
- Myxopapillary ependymoma	2	C72.0, C72.1	9394/1
- Papillary tumor of the pineal region	2	C75.3	9395/3
- Astrocytoma, NOS	2	C71, C72.0	9400/39
- Low grade astrocytoma	2	C71, C72.0	9400/32
- High grade/anaplastic astrocytoma	2	C71, C72.0	9401/33
- Desmoplastic infantile astrocytoma/desmoplastic infantile ganglioglioma	2	C71	9412/1
- Dysembryoplastic neuroepithelial tumor	2	C71	9413/0
- Pilocytic astrocytoma	2	C71, C72.0	9421/1
- Optic nerve glioma, optic chiasm glioma in children	2	C72.3	9421/1
- Glioblastoma	2	C71, C72.0	9440/3
- Oligodendroglioma, NOS	2	C71	9450/39
- Low grade oligodendroglioma	2	C71	9450/32
- High grade/anaplastic oligodendroglioma	2	C71	9451/33
- Medulloblastoma, NOS	2	C71.6	9470/3
- Embryonal tumor of the CNS, NOS	2	C71, C72.0	9473/3
- Gangliocytoma	2	C71, C72.0, 75.1	9492/0
- Dysplastic gangliocytoma of the cerebellum	2	C71.6	9493/0
- Ganglioglioma	2	C71, C72.0	9505/1
- Neurocytoma	2	C71	9506/1
- Multinodular and vacuolating neuronal tumor	2	C71	9509/0
- Glioneural tumor	2	C71, C72.0	9509/1
- Meningioma, NOS	2	C70	9530/0
- Atypical meningioma	2	C70	9539/1
- Anaplastic (malignant) meningioma	2	C70	9530/3
- Schwannoma	2	C72.4, C72.5	9560/0
Hematological malignancies			
- Primary lymphoma of the central nervous system	2	C71	9590/3
- Langerhans cell histiocytosis	2	C34, C41, C71^***^	9751/3

Other specific cancers not listed here may be diagnosed through clinical investigations. A specific morphology code should only be applied after evaluation by a coding expert of the cancer registry.

NOS, not otherwise specified.

^*^ Sarcomas can be localized at any site, but mostly occur in the soft tissues, including the retroperitoneum and the mediastinum.

^**^ Angiosarcoma of the (skin of the) breast following radiotherapy of the breast.

^***^ Other sites are possible.

**Table 4** presents a roster of cancers that can be diagnosed using elevated tumor markers in conjunction with clinical investigations and when pathology is not available.

**Table 4:** Cancers that can be diagnosed based on an elevated tumor markers in combination with clinical investigations.

Cancer type	Tumor marker	ICD-O Morphology code
Colorectal cancer	Carcinoembryonic antigen (CEA)	8000/3
Hepatocellular carcinoma	Alfa-fetoprotein (AFP)	8170/3
Pancreatic cancer, cancer of the gallbladder/bile ducts	Cancer antigen 19-9 (CA 19-9)	8000/3
Ovarian cancer	Cancer antigen 125 (CA-125)	8000/3
Prostate cancer	Prostate-specific antigen (PSA)	8000/3
Choriocarcinoma of the placenta	Human chorionic gonadotropin (HCG)	9100/3
Germ cell tumor	Human chorionic gonadotropin (HCG)	9064/3
	Alfa-fetoprotein (AFP) (+/- HCG)	9065/3
Neuroendocrine tumor	Chromogranin A	8240/3
Functioning neuroendocrine tumors (excluding pituitary gland tumors)	Insulin Glucagon Gastrin Vasoactive intestinal peptide (VIP) Somatostatin Serotonin Adrenocorticotropic hormone (ACTH) and other hormones	8151/3 8152/3 8153/3 8155/3 8156/3 8241/3 8158/3
Medullary thyroid carcinoma	Calcitonin	8345/3
Neuroblastoma	Catecholamine degradation products (Homovanilic acid [HVA], Vanillylmandelic acid [VMA])	9500/3
Prolactinoma	Prolactin	8271/0
Other functioning pituitary gland tumors	Growth hormone, Follicle-stimulating hormone (FSH), Luteinizing hormone (LH), Adrenocorticotropic hormone (ACTH), thyroid stimulating hormone (TSH)	8272/0
Phaeochromocytoma	Catecholamines, Chromogranin A	8700/3
Multiple myeloma	M-protein (IgG, IgM, IgA) >30g/L	9732/3
Waldenström’s macroglobulinemia	IgM	9761/3

When utilizing these tables in registration practice, the following rules should be observed.

1) Use the highest code from the range 1-8 (**Table 1**), unless it is a ‘death certificate only’ (DCO) case (basis of diagnosis 0) or if the basis of diagnosis cannot be determined (basis of diagnosis 9).

The order of the codes for the basis of diagnosis (from 1 to 8) represents an increasing reliability of the cancer diagnosis. The highest code within the range should be assigned to represent the most reliable basis of diagnosis.

2) Use code 0 when trace back from the death certificate is not possible. DCO cases should be registered with morphology code 8000, unless the morphology code can be derived from the ICD code (C43 [8720/3], C45 [9050/3], C46 [9140/3], and C81-C96/D45-D47 [9590/3-9989/3]) or from the text on the death certificate (e.g., ‘adenocarcinoma of the stomach’ or ‘rhabdomyosarcoma’).

Limited information is generally available for DCO cases, but even with only a coded cause of death, the morphology can be deducted in several instances. Some registries have access to detailed information on the death certificate, which should be used for morphology coding if available.

3) Code 1 should only be used for cancers that are detected by physical examination only. This includes cancers of head and neck, eye, breast, skin and superficial soft tissues, external genitals, vagina, cervix, anus, rectum, and prostate. It is almost impossible to diagnose a cancer in most inner organs (such as the lung, stomach, colon, or kidney) with physical examination only, but rare exceptions are possible.

Only a few cancers may be diagnosed with physical examination alone. As physical examination is typically followed by a biopsy and/or imaging in most cases, the number of cases with physical examination as the basis of diagnosis is very small.

4) Codes 1 and 2 may be used when a diagnosis of cancer is at least **likely** (‘probably cancer’). If clinical investigations reveal that a cancer diagnosis is possible, the case should not be registered in the absence of pathological confirmation (basis of diagnosis 5-8).

To avoid overestimating the number of cancers, cases should only be registered when the symptoms or appearances are most likely caused by cancer. If multiple disorders, including cancer, could explain the symptoms or appearances, the case should not be registered. For example, if the diagnosis includes ‘large lesion in the left cerebellum, differential diagnosis arterial malformation, low grade neuronal tumor’ the case should not be registered, as a non-malignant disorder could also explain the symptoms or appearances.

5) Cancers registered with basis of diagnosis 1 or 2 should be assigned morphology code 8000/3 (8000/0 or 8000/1 are also allowed for benign and borderline malignant tumors of the central nervous system). Exceptions to this rule are listed in **Table 3**. These exceptions apply only to cases where a specific diagnosis is at least **likely**. If the diagnosis is only **possible** or multiple diagnoses are mentioned in the clinical file or report, the case should be registered with morphology code 8000/3 (8000/0 or 8000/1 are also allowed for benign and borderline malignant tumors of the central nervous system).

**Table 3** provides an overview of specific tumor entities that may be diagnosed using imaging or physical examinations. If the diagnosis in the clinical report is relatively certain, that specific diagnosis should be coded. For example, if the report states ‘lesion in the frontal lobe, typical for glioblastoma’, the morphology code of glioblastoma (9440/3) should be used in combination with basis of diagnosis 2 (clinical investigation). However, if the report states ‘low-grade lesion in the temporal lobe; differential diagnosis DNET, ganglioglioma, low-grade astrocytoma’ morphology code 8000/1 should be used.

6) Code 4 (specific tumor markers) should always be used in combination with a clinical diagnosis of cancer and/or a clinical investigation showing cancer since many tumor markers, such as prostate-specific antigen (PSA), may also be increased in the absence of cancer. The cancers that may be registered with basis of diagnosis 4 are listed in **Table 4**.

Although tumor markers may be increased in many cancers, they can also be increased in the absence of cancer. Therefore, when coding basis of diagnosis 4, it should always be accompanied by a clinical diagnosis (for example increased PSA in combination with a malignant appearance of the prostate at rectal examination) or a clinical investigation (for example increased alfa-fetoprotein in combination with LiRADS 6).

7) Flow cytometry is often used for the diagnosis of leukemia and lymphoma, such as chronic lymphocytic leukemia.

Flow cytometry is classified with the same code as cytology since it utilizes cell suspension.

8) If a genetic abnormality specific to cancer is found through ‘liquid’ biopsy (in combination with a clinical diagnosis of cancer, but in the absence of pathological confirmation), basis of diagnosis 5 should be applied.

A liquid biopsy involves detection of cancer cells or fragments of DNA from these cancer cells in blood or other body fluids. In cases where no pathological information is available, a liquid biopsy is classified with the same code as cytology.

9) Codes 7.1-7.3 are optional for cases with histology.

Although not necessary for international comparison, several registries may choose to distinguish various categories of histology: histology of the primary tumor, histology of a metastatic site, and histology at autopsy. While these categories are equal in terms of the certainty of the diagnosis, the different categories may be useful for other purposes, such as staging or cross-checks (e.g., coding histology from a metastatic site (7.2) means that the patient has metastatic disease).

10) Many tumors have genetic abnormalities, but only a few are specific to the diagnosis of a certain cancer. Basis of diagnosis 8 should be used only when the genetic abnormality is specific for that cancer. In most cases, the abnormality should be present (e.g., CML, BCR-ABL1+ is 9875/3), but there are also cancer diagnoses characterized by the absence of a genetic abnormality (e.g., glioblastoma IDH wild type is 9445/3). Basis of diagnosis 8 applies to both examples.

Our understanding of cancer cells and their genetic properties has improved significantly in recent decades. Specific genetic abnormalities have been identified in an increasing proportion of cancers, leading to the classification of cancer entities based on these abnormalities. While some cancers already have separate morphology codes for cases with and without cytogenetic/molecular confirmation, others do not.

Hence, basis of diagnosis 8 was introduced to distinguish cases with and without cytogenetic/molecular confirmation until specific morphology codes are available for cancer entities defined by genetic abnormalities. Basis of diagnosis 7 should be used for cases in which cytogenetic/molecular diagnostics were not performed, but a pathological diagnosis was available. This code may become obsolete in the future if specific morphology codes will become available for cancer entities that are defined by genetic abnormalities.

## Conclusion

The updated recommendations introduce an adapted code list for the basis of diagnosis and incorporate new techniques, while maintaining consistency with the original version. Consolidating histological codes and introducing a new code for cytogenetic/molecular confirmation enhances the accuracy and specificity of cancer diagnoses. Additionally, the inclusion of specific morphological codes based on clinical information or investigations improves the classification of cancers without pathological confirmation. These updates will have a minimal impact on cancer registry operations and contribute to reducing the number of cases with unspecified morphological codes, particularly for central nervous system tumors, leading to enhanced international comparability of cancer registry data.

## Data availability statement

The original contributions presented in the study are included in the article/supplementary material. Further inquiries can be directed to the corresponding author.

## Author contributions

All authors contributed to the development of the ENCR Recommendations. The first draft of the manuscript was written by OV. All authors critically reviewed and approved the final manuscript.
